# Extracellular vesicles in smoking-related lung diseases

**DOI:** 10.18632/oncotarget.6556

**Published:** 2015-12-10

**Authors:** Yu Fujita, Jun Araya, Takahiro Ochiya

**Affiliations:** Division of Molecular and Cellular Medicine, National Cancer Center Research Institute, Tokyo, Japan

**Keywords:** Chromosome Section, extracellular vesicle, exosome, autophagy, microRNA, COPD

Cigarette smoking continues to be a major health hazard, and is involved in the mechanisms of developing various types of smoking-related diseases. Chronic obstructive pulmonary disease (COPD) is a representative disease mainly caused by cigarette smoke inhalation and is pathologically characterized by emphysema and fibrotic airway remodeling. In addition, the presence of COPD is associated with higher incidence of lung tumors. Repeated smoke exposure damages the epithelial barrier and eventually induces excessive inflammation accompanied by phenotypic alteration of lung epithelial cells. Phenotypical change of lung epithelial cells may result in alteration of the finely balanced reciprocal interaction between the epithelial and mesenchymal cell types that influences cellular differentiation, which is known as the epithelial-mesenchymal trophic unit (EMTU). Aberrant reactivation of EMTU has been postulated to be responsible for COPD development, such as airway remodeling, indicating that cell-to-cell communication within the lung microenvironment is one of the focuses in smoking-related lung disease pathogenesis [1].

Extracellular vesicles (EVs), such as exosomes and microvesicles, have gained increasing interest because of their potential functions in intercellular communication *via* vesicle cargo [2]. The EV components, including DNA, mRNA, microRNA (miRNA), and proteins, are capable of influencing the multiple biological processes of the recipient cells, resulting from regulating gene expression levels. So far, scientists have elucidated the participation of EV-mediated reciprocal interactions of cell phenotypic alterations during COPD pathogenesis, which may represent an entirely new paradigm for pathogenic transcellular signaling.

Recently, we have reported a novel pathogenic mechanism for airway remodeling in COPD, which can be attributed to cigarette stress-induced EVs derived from primary human bronchial epithelial cells (HBECs) [3]. We found that HBEC-derived EVs in response to cigarette smoke extract (CSE) induce myofibroblast differentiation in primary lung fibroblasts (LFs). Thorough evaluations of the modified EVs and COPD lung specimens clarified that cigarette smoke-induced upregulation of miR-210 expression in EV from HBECs is responsible for this profibrotic phenotypic change in LFs. Remarkably, miR-210 negatively regulates autophagy processes and downregulates ATG7, an essential component of autophagy machinery, in LFs. Silencing ATG7 in LFs confirmed participation of autophagy in miR-210-mediated myofibroblast differentiation. Finally, we demonstrated that miR-210 was highly expressed in airway epithelial cells in COPD lungs and autophagy activity was significantly decreased in LFs from COPD patients. These data suggest that transferred EV-mediated autophagy suppression as a part of the mechanisms for EMTU reactivation might be involved in the mechanisms for airway remodeling during COPD pathogenesis.

Autophagy is a conserved process of lysosomal self-degradation that helps to maintain the homeostatic balance between the synthesis, degradation and recycling of cellular components. Accordingly, it is not surprising that physiological EV secretion may function in close relation with the autophagy pathway to preserve protein and RNA homeostasis, and to mediate the spreading of signals to surrounding cells in order to coordinate organismal systemic responses [4]. Besides physiological autophagy regulation, we consider that EV transfer might be a crucial regulator of the aberrant autophagy process during disease pathogenesis. Indeed, Dutta et al. reported that breast tumor cell-derived EVs are taken up by human mammary epithelial cells (HMECs), resulting in the induction of reactive oxygen species and autophagy [5]. In addition, the autophagy modulation by EVs was associated with releasing breast tumor cell growth promoting factors from HMECs in the tumor microenvironment. Recently, Phinney et al. reported that mesenchymal stem cells (MSCs) use EVs to outsource mitophagy and shuttle miRNAs [6]. They showed that MSC-derived EV miRNAs facilitated macrophages to engulf and re-utilize transferred mitochondria from MSCs, further supporting the notion of crucial involvement of EVs in paracrine regulation of autophagy/mitophagy. Therefore, it is likely that further understanding of the autophagy machinery regulated by EVs will open a new path for elucidating disease pathogenesis.

Additionally, as a part of supplementary data of our recent work, we showed that 6 members of the *let-7* family (*let-7a*, *let-7b*, *let-7c*, *let-7f*, *let-7g*, and *let-7i*) were significantly downregulated in HBEC-derived EVs after CSE exposure [3]. In lung tumors, the expression levels of the *let-7* family members are generally reduced compared with those in normal lung tissues [7]. These tumor suppressive miRNA alterations have been recognized to contribute to lung tumor development, suggesting a potential causal link between reduction of *let-7* family in EVs and lung carcinogenesis in response to cigarette smoke exposure *via* EV transfer communication of these pathological cells.

Thus, bronchial epithelial cell-derived EVs modulated by cigarette smoke exposure may have a crucial pathogenic role in the development of various smoking-related lung diseases, including COPD and lung tumors. Further analysis of the contributing mechanisms of EVs and the alterations in EV content derived from pathological cells during disease progression may help us not only to understand the mechanisms but also to develop novel treatment strategies.
